# Integrating machine learning and clinicopathological data to stratify survival risk in young women with localized breast cancer

**DOI:** 10.3389/fmed.2026.1793790

**Published:** 2026-05-05

**Authors:** Bin Xu, Jun Shen, Jianguo Shen

**Affiliations:** The Sir Run Run Shaw Hospital, Affiliated to Zhejiang University School of Medicine, Hangzhou, Zhejiang, China

**Keywords:** machine learning, precision oncology, Random Survival Forest, risk stratification, young-onset breast cancer

## Abstract

**Background:**

Young women with localized breast cancer represent a clinically distinct population with heterogeneous outcomes, yet age-specific prognostic models remain limited. Conventional risk stratification tools derived from mixed-age cohorts may fail to capture the complex interactions between tumor biology and treatment response in this group.

**Methods:**

We conducted a single-center retrospective cohort study including 1,060 women aged ≤40 years diagnosed with stage I–III breast cancer between 2000 and 2023. Overall survival (OS) was analyzed using Kaplan–Meier estimates and multivariable Cox regression. To enable data-driven risk prediction beyond linear assumptions, a machine learning–based Random Survival Forest (RSF) model was developed to identify key prognostic features, quantify variable importance, and stratify patients into distinct risk groups.

**Results:**

Among 1,060 eligible patients, 110 deaths (10.4%) occurred during a median follow-up of 79.8 months. Invasive pathological subtype (hazard ratio [HR] = 5.23, 95% confidence interval [CI] 1.18–23.22; *p* = 0.030), nipple invasion (HR = 3.95, 95% CI 2.14–7.27; *p* < 0.001), and advanced T stage (T2-4 vs. Tis/T1; HR = 1.60, 95% CI 1.03–2.48; *p* = 0.036) were independently associated with worse OS. By contrast, receipt of endocrine therapy (HR = 0.54, 95% CI 0.36–0.80; *p* = 0.002) and radiotherapy (HR = 0.53, 95% CI 0.32–0.86; *p* = 0.010) were associated with better OS. Notably, high Ki67 expression (≥35%; HR = 0.39, 95% CI 0.21–0.71; *p* = 0.002) was associated with improved OS. The RSF model confirmed these predictors, ranked radiotherapy as the most influential variable, and provided effective risk stratification (C-index = 0.723).

**Conclusion:**

By integrating clinicopathological variables with machine learning–based survival modeling, this study identified key prognostic factors associated with OS in young women with localized breast cancer. The findings highlight the prognostic importance of treatment-related factors and reveal an unexpected association between high Ki-67 expression and better survival in this population. These data-driven risk stratification approaches may contribute to more personalized prognostic assessment and warrant validation in prospective multicenter studies.

## 1 Introduction

Breast cancer remains the most prevalent malignancy among women worldwide and continues to impose a significant public health burden (1–3). Although advances in early detection, surgical techniques, and systemic therapies have markedly improved patient survival, disease recurrence, distant metastasis, and therapeutic resistance persist as major challenges (4–6). These limitations highlight the need for further refinement in prognostic assessment and the development of more personalized treatment strategies.

Young-onset breast cancer, conventionally defined as diagnosis at or before 40 years of age, displays distinct clinicopathological and biological characteristics compared with breast cancer in older women (7). Although representing a smaller proportion of overall cases, tumors in this subgroup are more likely to exhibit aggressive features, including higher frequencies of triple-negative or HER2-positive subtypes, more advanced stage at diagnosis, elevated proliferation indices such as Ki67, and potentially unique molecular signatures (8–12). Clinical decision-making in young patients is further complicated by the need to consider fertility preservation, long-term quality of life, and late treatment-related toxicities. Therefore, early identification of high-risk individuals and timely implementation of intensive therapeutic interventions are essential. Existing prognostic models, which are largely developed from mixed-age patient cohorts, may not adequately reflect the unique determinants of outcome in younger patients (13–15). This limitation creates a clear evidence gap in risk stratification and in the design of treatment strategies specifically tailored to this population.

To address this gap, we conducted a single-center, retrospective cohort study of young patients with localized breast cancer. This study aimed to comprehensively characterize the clinicopathological profile of this population and to identify independent prognostic factors associated with overall survival (OS) by employing an integrated analytical strategy that combined traditional multivariable regression with a machine-learning-based Random Survival Forest (RSF) model. The findings are intended to provide more nuanced evidence to inform precise risk stratification and individualized management for young patients with breast cancer.

## 2 Methods

### 2.1 Study design and participants

This retrospective study was done at Sir Run Run Shaw Hospital. We screened all consecutive patients diagnosed with breast cancer between January 1st, 2000, and December 31st, 2023. The inclusion criteria were as follows: (1) aged at diagnosis ≤40 years; (2) newly diagnosed with localized (stage I-III) breast cancer according to the 8th edition of the American Joint Committee on Cancer (AJCC) staging system; (3) underwent curative-intent surgical resection as the primary treatment. Patients were excluded if they met any of the following criteria: (1) male gender; (2) combined with other malignancies; (3) incomplete clinical, pathological, treatment, or follow-up records for key variables with substantial missing data (defined as >10% missing).

This study was approved by the Ethics Committee of the Sir Run Run Shaw Hospital, affiliated with Zhejiang University School of Medicine. The requirement for informed consent was waived due to the retrospective nature of the study, as all data were anonymized before analysis, and the research involved no more than minimal risk to participants. The median follow-up time was 79.8 months (interquartile range, IQR: 37.2–136.3 months), and a total of 110 deaths (10.4%) occurred among the 1,060 patients during the study period. A total of 950 patients (89.6%) were censored.

### 2.2 Procedures and study outcomes

Comprehensive demographic, clinical, pathological, and treatment strategies were retrieved from the hospital’s electronic medical record system. The following variables were extracted: age at diagnosis, menopausal status, TNM stage, histological type, status of lymphovascular invasion, perineural invasion, and nipple involvement. Standard immunohistochemistry was used to assess biomarker status. ER and PR positivity were defined as the presence of nuclear staining in ≥1% of tumor cells. HER2 status was considered negative for immunohistochemistry (IHC) scores of 0 or 1+, and positive for a score of 3+. Cases with an IHC score of 2 + were further evaluated using fluorescence *in situ* hybridization (FISH) for final determination. Treatment details included: type of surgery (breast-conserving surgery or mastectomy), and the receipt of any adjuvant radiotherapy, chemotherapy, endocrine therapy, and targeted therapy. The primary endpoint for this study was OS, defined as the time from the date of histological diagnosis to the date of death from any reason or the last follow-up.

### 2.3 Statistical analysis

All statistical analyses were performed using R software (version 4.5.1). Continuous variables were presented as the mean (standard deviation, SD) if they were normally distributed or as the median (IQR) if not. Categorical variables were presented as numbers (percent). For variables with low rates of missing data (<5%), multiple imputation by chained equations was performed using the ‘mice’ package in R. Five imputed datasets were generated, and results were pooled according to Rubin’s rules. Variables with substantial missing data (>10%) were excluded from the analysis, and patients with missing data for these variables were not included in the cohort. Survival curves were estimated using the Kaplan–Meier method and compared with the log-rank test. Univariate and multivariable Cox proportional hazards regression analyses were performed using the ‘survival’ package (version 3.5–5) in R. Univariate Cox proportional hazards regression was used to assess the association between variables and OS. All variables with a *p* value less than 0.05 in the univariate analysis were entered into a multivariable Cox proportional hazard regression model to identify independent prognostic factors. Results were reported as hazard ratios (HR) with 95% confidence interval (CI). A two-sided *p* < 0.05 was considered statistically significant.

To complement the traditional regression approach and account for potential complex, non-linear relationships among variables, a machine learning-based RSF analysis was subsequently performed using the randomForestSRC package in R. The model was constructed using all clinicopathological variables as inputs, with the survival outcome defined by OS time and status. Key hyperparameters were set based on standard recommendations in the literature and preliminary experimentation: the number of trees (ntree) was set to 1,000 to ensure stability of the results, the splitting rule was “logrank” as it is the default and most widely used rule for survival outcomes, and the minimum terminal node size (nodesize) was set to 15 to avoid overfitting while maintaining sufficient events per node (16). Formal hyperparameter optimization (e.g., grid search) was not performed, as our primary focus was on variable importance and risk stratification rather than maximizing predictive accuracy; moreover, random forest algorithms are known to be relatively robust to hyperparameter choices within reasonable ranges (17). The model’s predictive performance was evaluated using the out-of-bag (OOB) concordance index (C-index). OOB evaluation is an inherent feature of random forest algorithms that provides unbiased internal validation by using the approximately one-third of observations not included in each bootstrap sample, offering the advantage of efficient sample utilization without requiring a separate validation set. The relative importance of each variable for prediction was quantified using the Variable Importance (VIMP) metric. Finally, based on the individualized risk scores predicted by the RSF model, patients were stratified into high- and low-risk groups using the median risk score as the cutoff point for subsequent Kaplan–Meier survival analysis.

To account for potential temporal changes in diagnostic and treatment practices over the long study period, we additionally included diagnosis year as a categorical covariate (grouped into five intervals: 2000–2004, 2005–2009, 2010–2014, 2015–2019, and 2020–2023) in the multivariable Cox model as a sensitivity analysis.

## 3 Results

### 3.1 Clinicopathological characteristics of the patients

**Figure 1:**
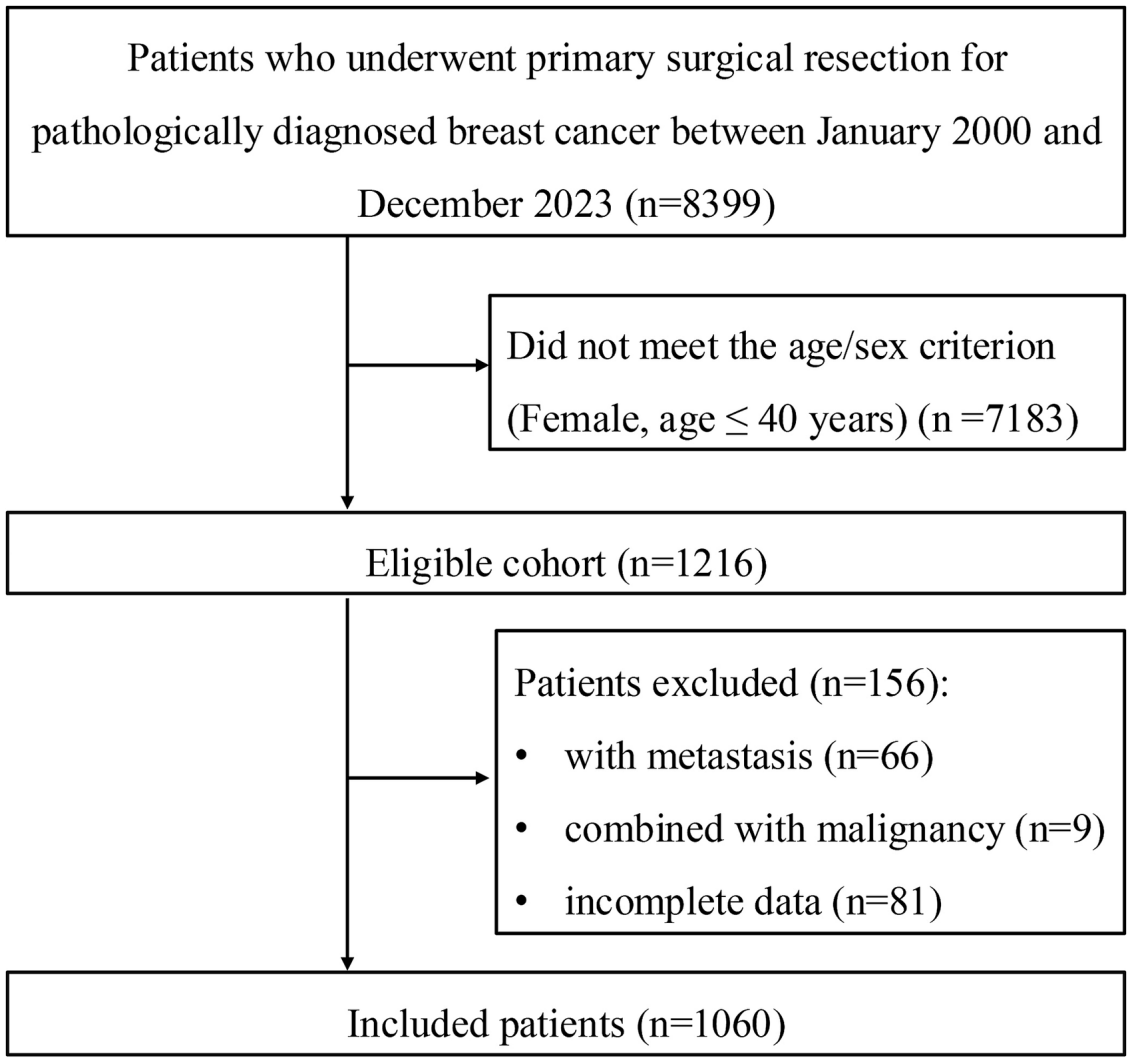
Flowchart of patient inclusion and exclusion.

Between January 2000 and December 2023, a total of 1,060 patients met our criteria and were enrolled in our final analysis (see Figure 1). The baseline clinicopathological characteristics of the study population are summarized in Table 1. The median age at diagnosis was 36 years (IQR: 33–39), and only 35 patients (3.3%) were postmenopausal. Regarding tumor stage at diagnosis, 565 patients (53.3%) were classified as clinical T1 and 356 (33.6%) as T2, while 89 (8.4%) had carcinoma *in situ* (Tis). Advanced T stages (T3 and T4) accounted for 3.3 and 1.4% of cases, respectively. Consistent with the T stage distribution, most patients (864, 81.5%) were clinically lymphnode-negative (N0).

**Table 1:** Baseline characteristics of the included patients (*N* = 1,060).

Characteristics	*N* (%)/median (IQR)
Age, years	36 (33–39)
Menopausal status	
Premenopausal	1,025 (96.7%)
Postmenopausal	35 (3.3%)
T stage	
Tis	89 (8.4%)
T1	565 (53.3%)
T2	356 (33.6%)
T3	35 (3.3%)
T4	15 (1.4%)
N stage	
N0	864 (81.5%)
N1	129 (12.2%)
N2	56 (5.3%)
N3	11 (1.0%)
Type of surgery	
Total mastectomy	512 (48.3%)
Breast-conserving surgery	548 (51.7%)
Histological type	
Non-invasive	164 (15.5%)
Invasive	871 (82.2%)
Others	25 (2.4%)
ER positive	787 (74.2%)
PR positive	665 (62.7%)
HER2 positive	243 (22.9%)
Ki-67 index	25% (10–35%)
Lymphovascular invasion	60 (5.7%)
Perineural invasion	11 (1.0%)
Nipple involvement	29 (2.7%)
Chemotherapy	
No chemotherapy	314 (29.6%)
Neoadjuvant chemotherapy	189 (17.8%)
Adjuvant chemotherapy	557 (52.5%)
Radiotherapy	736 (69.4%)
Endocrine therapy	789 (74.4%)
Targeted therapy	120 (11.3%)

IQR, interquartile range; ER, estrogen receptor; PR, progesterone receptor; HER2, human epidermal growth factor receptor 2.

Surgically, a near equal distribution was observed between total mastectomy (512 patients, 48.3%) and breast-conserving surgery (548 patients, 51.7%). Histologically, invasive carcinoma constituted the predominant type (871 patients, 82.2%), followed by non-invasive carcinoma (164 patients, 15.5%). Analysis of key molecular markers revealed that hormone receptor-positive tumors were common: 787 patients (74.2%) were estrogen receptor (ER)-positive and 665 (62.7%) were progesterone receptor (PR)-positive. Human epidermal growth factor receptor 2 (HER2) positivity was identified in 243 patients (22.9%). The median Ki67 index was 25% (IQR: 10–35%). Pathological examination showed the presence of lymphovascular invasion in 60 patients (5.7%), perineural invasion in 11 (1.0%), and nipple involvement in 29 (2.7%).

In terms of treatment, a total of 746 patients (70.3%) underwent chemotherapy: 189 (17.8%) received neoadjuvant chemotherapy, and 557 (52.5%) received adjuvant chemotherapy, while 314 patients (29.6%) did not receive chemotherapy. Radiotherapy was administered to 736 patients (69.4%). Endocrine therapy, reflecting the high prevalence of hormone receptor-positive disease, was given to 789 patients (74.4%). Targeted therapy, primarily for HER2-positive disease, was utilized in 120 patients (11.3%).

### 3.2 Univariate and multivariate cox regression analysis

**Figure 2:**
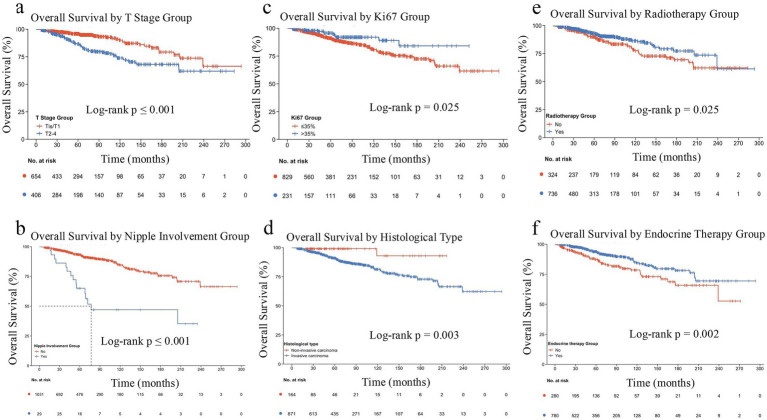
Kaplan–Meier estimates of overall survival. Survival curves are stratified by T stage **(a)**, nipple invasion **(b)**, Ki67 expression **(c)**, histological subtype **(d)**, radiotherapy **(e)**, and endocrine therapy **(f)**.

Univariate Cox analysis identified several factors significantly associated with OS (Table 2). Poorer OS was strongly associated with advanced tumor stage (T2-4 vs. Tis/T1; HR = 2.42, 95% CI: 1.64–3.57, *p* < 0.001), positive nodal status (N1-3 vs. N0; HR = 2.56, 1.74–3.77, *p* < 0.001), total mastectomy (vs. breast-conserving surgery; HR = 2.04, 1.33–3.12, *p* = 0.001), invasive pathology (vs. non-invasive; HR = 6.37, 1.57–25.82, *p* = 0.010), and nipple invasion (HR = 4.45, 2.58–7.70, *p* < 0.001). Factors associated with better OS included older age (>32 vs. ≤32 years; HR = 0.61, 0.40–0.93, *p* = 0.020), high Ki-67 (>35%; HR = 0.52, 0.29–0.93, *p* = 0.028), and receipt of radiotherapy (HR = 0.65, 0.45–0.95, *p* = 0.026) or endocrine therapy (HR = 0.55, 0.38–0.80, *p* = 0.002). Compared to no chemotherapy, both preoperative (HR = 3.87, 2.03–7.38, *p* < 0.001) and postoperative chemotherapy (HR = 2.03, 1.09–3.79, *p* = 0.026) were linked to increased mortality (see Figure 2).

**Table 2:** Univariate Cox regression analysis of overall survival.

Variables	HR (95% CI)	*p*-value
Menstrual status (postmenopausal vs. premenopausal)	1.71 (0.91–3.22)	0.094
Age (>32 vs. ≤32 years)	0.61 (0.40–0.93)	**0.020**
T stage (T2-4 vs. Tis/T1)	2.42 (1.64–3.57)	**<0.001**
N stage (N1-3 vs. N0)	2.56 (1.74–3.77)	**<0.001**
Surgery (total mastectomy vs. breast-conserving surgery)	2.04 (1.33–3.12)	**0.001**
Pathology		
Non-invasive type	Reference	
Invasive type	6.37 (1.57–25.82)	**0.010**
Others	2.48 (0.22–27.33)	0.459
HoR (positive vs. negative)	0.73 (0.49–1.09)	0.128
HER2 (positive vs. negative)	1.20 (0.79–1.83)	0.387
Ki67 (>35% vs. ≤35%)	0.52 (0.29–0.93)	**0.028**
Lymphovascular invasion (yes vs. no)	1.22 (0.59–2.50)	0.593
Perineural invasion (yes vs. no)	1.37 (0.34–5.57)	0.657
Nipple invasion (yes vs. no)	4.45 (2.58–7.70)	**0.001**
Chemotherapy		
No chemotherapy	Reference	
Preoperative chemotherapy	3.87 (2.03–7.38)	**<0.001**
Postoperative chemotherapy	2.03 (1.09–3.79)	**0.026**
Radiotherapy (yes vs. no)	0.65 (0.45–0.95)	**0.026**
Endocrine therapy (yes vs. no)	0.55 (0.38–0.80)	**0.002**
Targeted therapy (yes vs. no)	0.82 (0.38–1.78)	0.622

HR, hazard ratio; C, confidence interval; HoR; hormone receptor; HER2, human epidermal growth factor receptor 2.

Bold font represents statistical significance.

**Figure 3:**
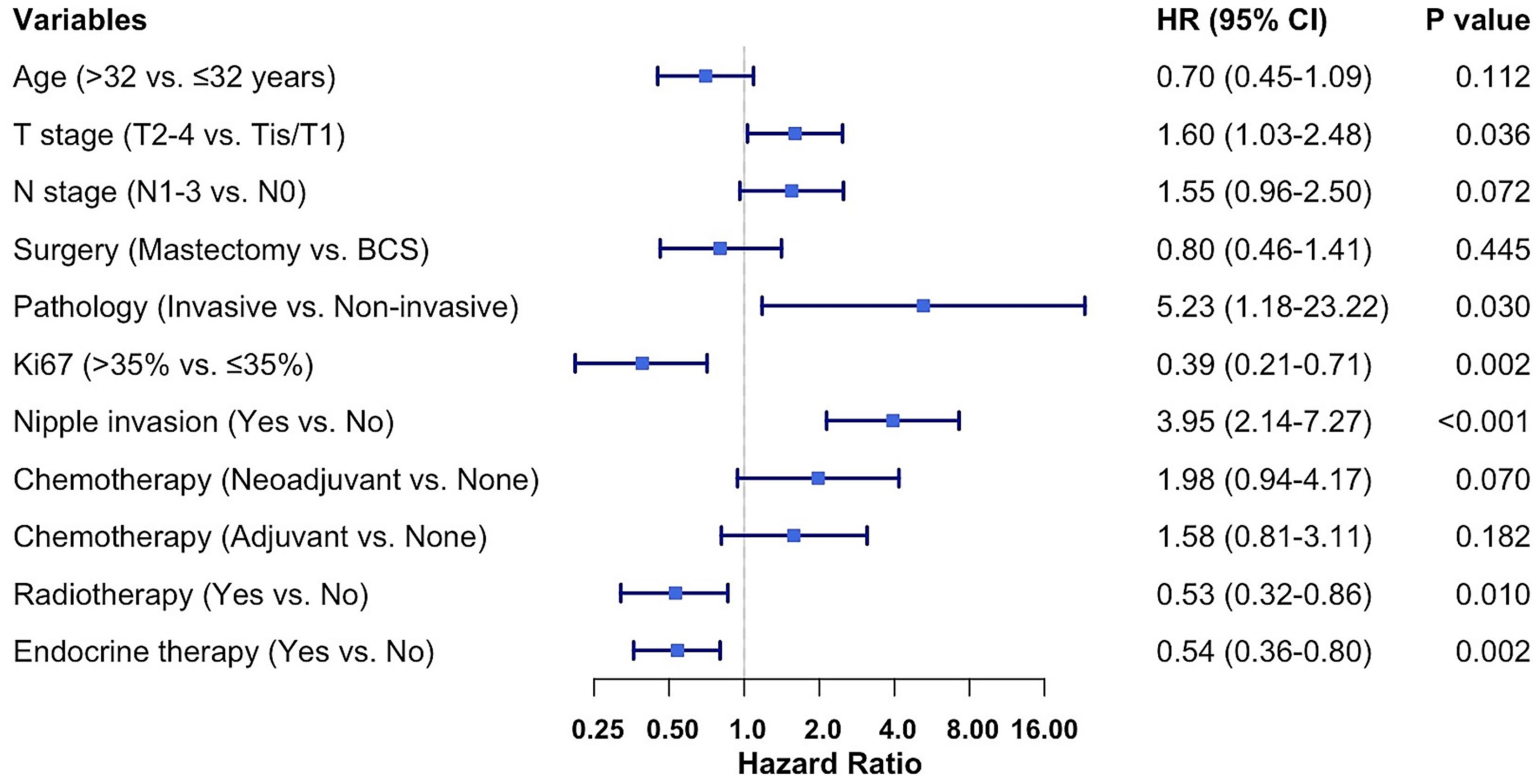
Multivariable Cox regression analysis of overall survival. The forest plot shows adjusted hazard ratios for the prognostic factors included in the final model. Variables associated with worse survival appear to the right of the vertical line (HR > 1), and those associated with better survival to the left (HR < 1). HR, hazard ratio; CI, confidence interval; BCS, breast conserving surgery.

Multivariate Cox regression analysis was performed to identify independent prognostic factors for OS (as shown in Figure 3). After adjusting for covariates, several variables remained significantly associated with OS. Poorer OS was independently predicted by invasive pathology (vs. non-invasive; HR = 5.23, 95% CI: 1.18–23.22, *p* = 0.030), nipple invasion (HR = 3.95, 95% CI: 2.14–7.27, *p* < 0.001), and advanced T stage (T2-4 vs. Tis/T1; HR = 1.60, 95% CI: 1.03–2.48, *p* = 0.036). Conversely, better OS was independently associated with high Ki67 expression (>35% vs. ≤35%; HR = 0.39, 95% CI: 0.21–0.71, *p* = 0.002), receipt of endocrine therapy (HR = 0.54, 95% CI: 0.36–0.80, *p* = 0.002), and radiotherapy (HR = 0.53, 95% CI: 0.32–0.86, *p* = 0.010). The final multivariable Cox model demonstrated good discriminative ability, with a bootstrap-corrected Harrell’s C-index of 0.712 (95% CI: 0.682–0.742).

In the sensitivity analysis adjusting for diagnosis year (grouped into five intervals), the results remained essentially unchanged (see Supplementary material), and diagnosis year itself was not significantly associated with OS, suggesting that temporal trends did not materially influence the main findings.

### 3.3 Complementary prognostic assessment via machine learning

To account for potential non-linear relationships and complex interactions among variables that may not be fully captured by the Cox model, an RSF analysis was performed using the same set of clinicopathological variables. The RSF model demonstrated good predictive performance, with an OOB C-index of 0.723 and an OOB error rate of 0.277. This C-index was comparable to that of the Cox model (0.712), suggesting that both approaches provided similar overall predictive accuracy in this cohort.

**Figure 4:**
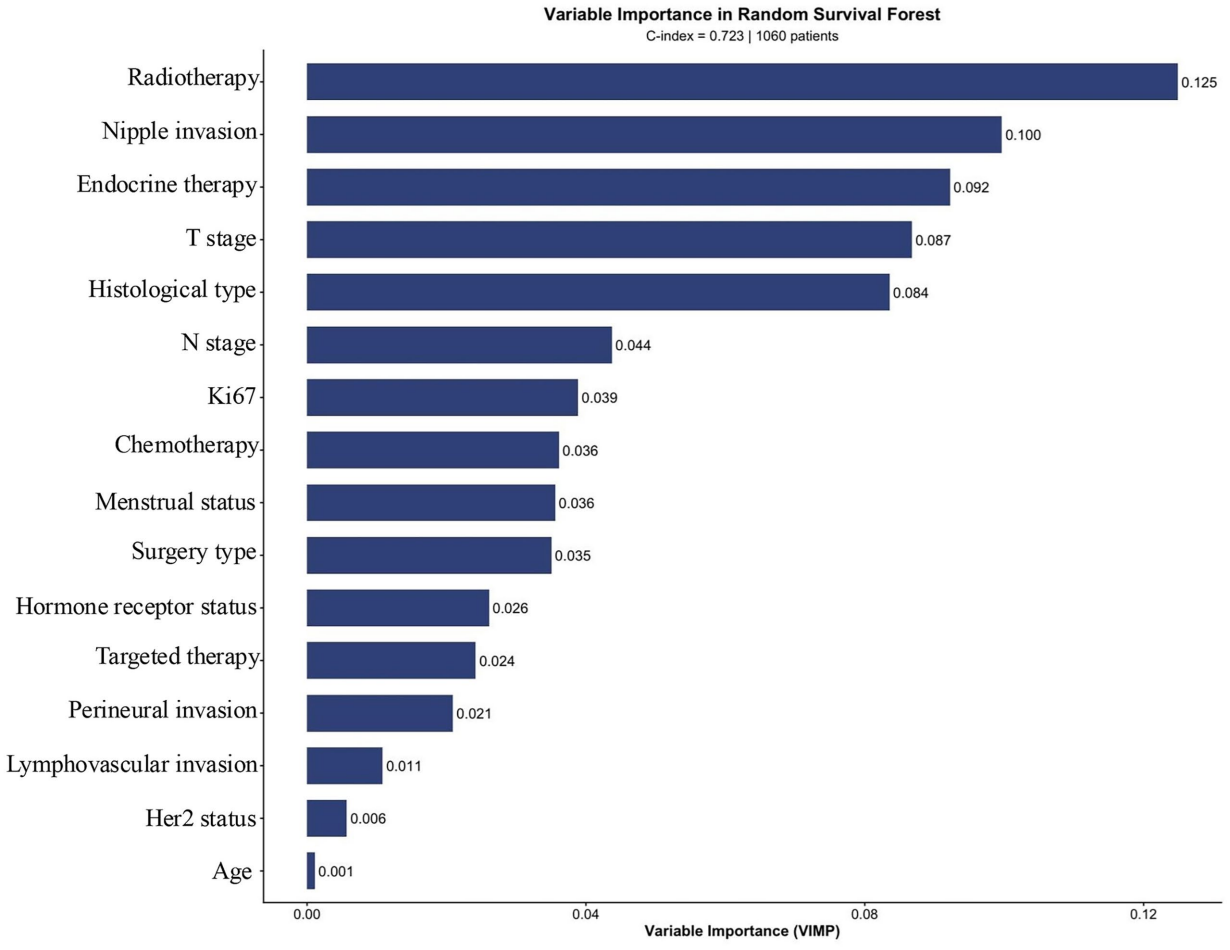
Variable importance plot from Random Survival Forest analysis. Variables are ranked by their contribution to survival prediction as measured by Variable Importance (VIMP).

The relative importance of each variable for prediction, as measured by the VIMP metric, is presented in Figure 4. Treatment-related factors, notably radiotherapy (VIMP = 0.125) and endocrine therapy (VIMP = 0.092), were among the top contributors to the model’s predictive accuracy. Nipple invasion (VIMP = 0.100) also ranked highly, consistent with its strong association with poor prognosis in the Cox model. Interestingly, while T stage and pathological subtype showed moderate importance in the RSF model, Ki67 expression—which was a significant protective factor in the Cox regression—demonstrated a comparatively lower ranking (VIMP = 0.039). Age had a minimal contribution to the RSF prediction (VIMP = 0.001).

**Figure 5:**
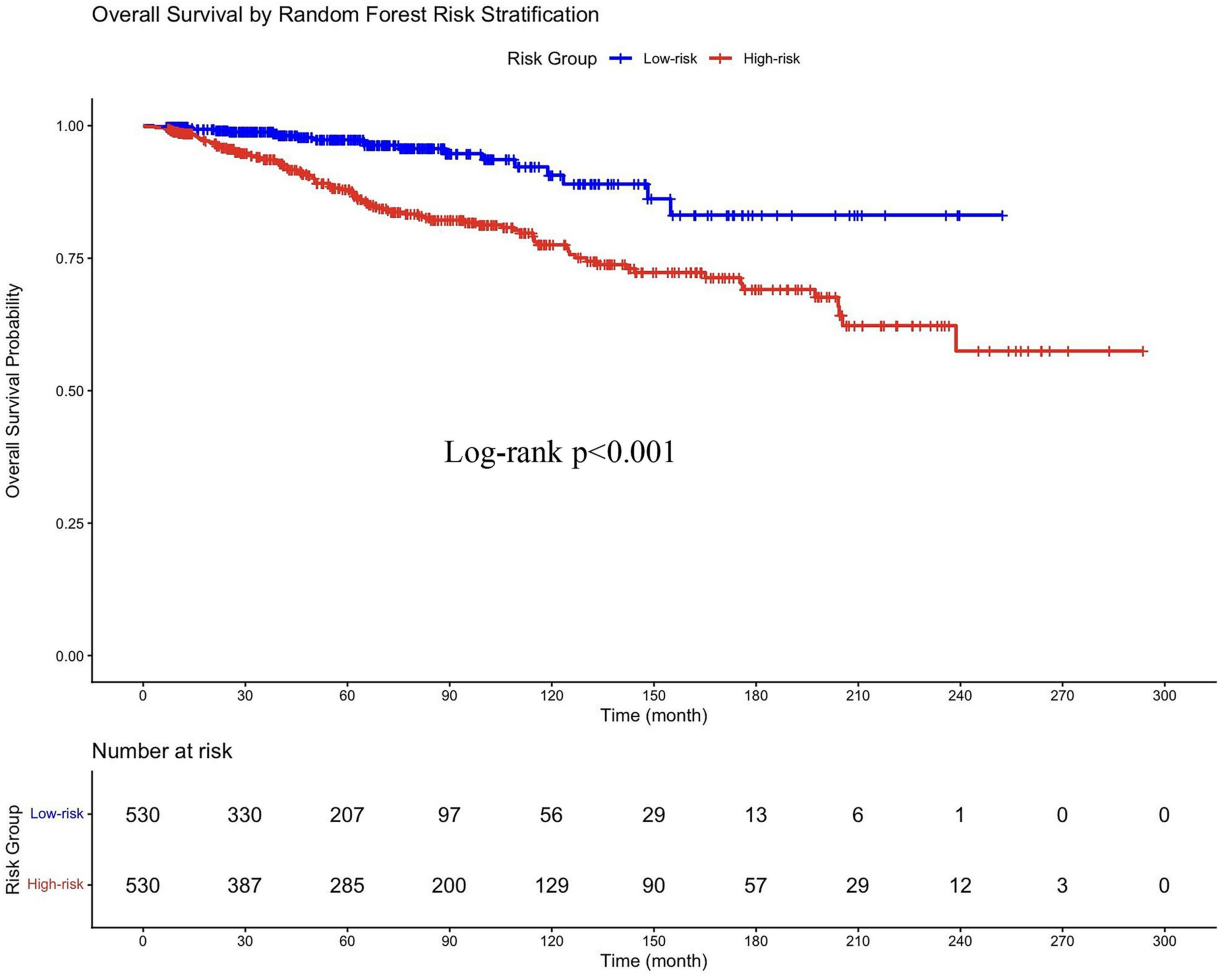
Kaplan–Meier survival curves stratified by Random Survival Forest (RSF) risk groups. Patients were classified into high-risk and low-risk groups based on median risk scores derived from the RSF model.

Using the individualized risk scores generated by the RSF model, the entire cohort was stratified into two groups: high-risk (*n* = 530) and low-risk (*n* = 530), based on the median risk score. Kaplan–Meier analysis confirmed a significant divergence in survival between these two groups (log-rank *p* < 0.001), with patients in the high-risk group experiencing markedly poorer OS (Figure 5). This effective risk stratification validates the RSF model’s capacity to synthesize multiple variables into a clinically meaningful prognostic tool.

### 3.4 Association between Ki67 expression and adjuvant treatment administration

To investigate whether the observed association between high Ki67 expression and better survival might be explained by differences in treatment patterns, we compared the receipt of adjuvant therapies between patients with high Ki67 (>35%) and low Ki67 (≤35%). As shown in Table 3, significant differences were observed across all treatment modalities. Patients with high Ki67 expression were more likely to receive chemotherapy (adjuvant: 75.3% vs. 46.2%; neoadjuvant: 15.6% vs. 18.5%; none: 9.1% vs. 35.3%; overall *p* < 0.001) and radiotherapy (79.7% vs. 66.6%, *p* < 0.001). They were also more likely to receive targeted therapy (17.7% vs. 9.5%, *p* = 0.001). Conversely, patients with high Ki67 were significantly less likely to receive endocrine therapy (51.5% vs. 79.7%, *p* < 0.001). These findings suggest that the survival benefit associated with high Ki67 may be partially mediated by more intensive use of chemotherapy, radiotherapy, and targeted therapy, despite lower receipt of endocrine therapy in this subgroup.

**Table 3:** Association between Ki67 expression and adjuvant treatment administration.

Treatment		Ki67 ≤ 35% (*n* = 829)	Ki67 > 35% (*n* = 231)	*p*-value
Chemotherapy	None	293 (35.3%)	21 (9.1%)	**<0.001**
	Neoadjuvant	153 (18.5%)	36 (15.6%)	
	Adjuvant	383 (46.2%)	174 (75.3%)	
Radiotherapy	No	277 (33.4%)	47 (20.3%)	**<0.001**
	Yes	552 (66.6%)	184 (79.7%)	
Endocrine therapy	No	168 (20.3%)	112 (48.5%)	**<0.001**
	Yes	661 (79.7%)	119 (51.5%)	
Targeted therapy	No	750 (90.5%)	190 (82.3%)	0.001
	Yes	79 (9.5%)	41 (17.7%)	

Bold font represents statistical significance.

## 4 Discussion

This study presents a comprehensive analysis of clinicopathological characteristics and identifies key prognostic factors associated with OS in young women with localized breast cancer. Invasive pathology, nipple invasion, and advanced T stage were independently associated with poorer OS, while high Ki67 expression, endocrine therapy, and radiotherapy emerged as factors associated with better OS. Notably, the machine learning-based Random Survival Forest model not only validated these findings by identifying radiotherapy, nipple invasion, and endocrine therapy as the strongest predictors, but also provided a robust risk stratification that effectively distinguished high- and low-risk patient groups.

The comparable predictive performance between the Cox model (C-index: 0.712) and the RSF model (C-index: 0.723) could suggest that the underlying relationships in this cohort are well-represented by linear assumptions. However, it is also highly plausible that this similarity in performance is a byproduct of the relatively low event rate (10.4%) observed in our study. Since machine learning algorithms like RSF typically require a substantial number of events to confidently map complex, non-linear interactions, the limited event density may have constrained the model’s ability to uncover more intricate patterns, leading to a performance level similar to traditional regression. Nevertheless, the RSF model still offered significant complementary value by ranking variable importance. This approach highlighted the dominant role of treatment-related factors—such as radiotherapy and endocrine therapy—which may be partially masked in the regression framework due to collinearity with tumor characteristics. Together, these results underscore the complex interplay between tumor biology and therapeutic interventions, demonstrating the benefit of combining hypothesis-driven regression with data-driven modeling in prognostic research.

Invasive pathological patterns and higher T stages are established indicators of more aggressive tumor biology and extensive local invasion. These characteristics are intrinsically linked to increased tumor burden and a higher potential for metastasis, which collectively contribute to significantly poorer clinical outcomes and survival in affected patients (18). Of note, this study found that nipple invasion is also an independent poor prognostic factor, generally meaning that the tumor may exhibit a more aggressive local spread pattern or is more likely to metastasize via specific anatomical and lymphatic pathways, thereby increasing the risk of local recurrence and distant metastasis (19, 20), ultimately leading to a shortened OS. Furthermore, the study reaffirms the significant survival benefits associated with endocrine therapy and radiotherapy. These treatment modalities, which are cornerstone interventions in standard care protocols, were identified as robust protective factors in our analysis (21, 22). Our findings, derived from real-world clinical data, provide further validation of their essential role in improving OS. This highlights the crucial importance of adhering strictly to evidence-based treatment strategies in clinical practice to maximize patient outcomes.

A particularly notable and seemingly paradoxical finding in our study is the identification of high Ki67 expression as an independent factor associated with better OS. This contrasts with the conventional understanding that elevated Ki67, a marker of high proliferative activity, is generally associated with more aggressive tumor biology and poorer prognosis (23–25). Several factors may contribute to this unexpected observation, and caution is warranted in its interpretation. First, this finding may reflect underlying differences in molecular subtype distribution within our cohort. High Ki67 is a common feature in aggressive subtypes such as HER2-positive and triple-negative breast cancer, which are typically treated with more intensive systemic therapies including chemotherapy and, for HER2-positive disease, targeted therapy (26–29). Second, a high proliferation index may intrinsically confer greater sensitivity not only to chemotherapy but also to radiotherapy, as these treatments primarily target rapidly dividing cells (30, 31). Therefore, tumors with high Ki67 might exhibit a more favorable response to adjuvant therapies, ultimately translating into improved survival outcomes (23, 32). Third, changes in Ki67 measurement and interpretation over the long study period (2000–2023) may have introduced variability, as immunohistochemical assessment and cutoff definitions have evolved over time. Finally, the distinct biology of young-onset breast cancer—which often exhibits higher proliferative activity yet remains treatment-responsive—may contextualize this counterintuitive finding. Notably, the RSF analysis ranked Ki67 relatively low in variable importance, suggesting that its prognostic contribution may be modest when considered alongside treatment-related factors. This further supports the interpretation that the observed association may be mediated by treatment intensity or subtype-specific management rather than representing a direct biological effect. While our data suggest this intriguing possibility, given the observational nature of the study and the potential for residual confounding by subtype, treatment, and temporal changes in measurement, these findings should be considered hypothesis-generating. Further mechanistic and prospective studies are warranted to validate this relationship and elucidate the underlying biological context.

Several limitations of this study should be acknowledged. First, the retrospective, single-center study design may introduce inherent selection bias, limiting the applicability of the findings to a broader population. Second, although all cases were reviewed by experienced pathologists at our hospital, the definition and assessment of certain pathological factors may differ in clinical practice. This is particularly relevant for Ki67, for which immunohistochemical assessment and cutoff values may have varied over the long study period. Third, as this is an observational study, the associations between treatment and survival should be interpreted with caution. Although we adjusted for multiple clinicopathological variables, residual confounding by indication—such as more aggressive treatment being given to higher-risk patients—cannot be fully excluded. Therefore, these findings reflect real-world associations rather than causal effects. Fourth, the overall event incidence in our study cohort was relatively low, at approximately 10.4%. This limited absolute number of events may have restricted the statistical power of our multivariable Cox regression and constrained the RSF model’s ability to fully optimize its decision trees. Consequently, the lower event density might have prevented the machine learning algorithm from capturing complex, non-linear interactions that would be more apparent in a cohort with higher mortality, potentially explaining why the machine learning approach did not significantly outperform the conventional regression model. Fifth, the long study period spans more than two decades, during which diagnostic criteria, treatment paradigms, and pathological assessment methods have evolved. Although we adjusted for available covariates, unmeasured changes over time may have influenced the observed associations. Future studies with period-stratified analyses are needed to validate these findings. Finally, despite multivariate adjustments, some unmeasured confounding factors may still exist, such as detailed chemotherapy regimens, genetic profiles, and patient comorbidities, which could affect the results. Additionally, the Cox and RSF models were evaluated using different validation methods (bootstrap vs. OOB), which are both well-established internal validation approaches for their respective modeling frameworks. While this may limit direct performance comparison, our primary objective was to assess the consistency of identified prognostic factors across methods rather than to determine which model performed better. To further refine these findings, future studies with larger cohorts should employ a common independent test set to facilitate more robust comparative assessments. Additionally, we recommend that subsequent research evaluate alternative clinical endpoints, such as recurrence-free survival, in addition to OS. The inclusion of RFS would likely yield a higher absolute number of events, thereby enhancing statistical power and providing more robust data for the optimization of machine learning algorithms like RSF. Ultimately, prospective multicenter studies utilizing standardized protocols remain essential to validate and refine these prognostic associations.

To contextualize the methodological rigor and potential limitations of our modeling approach, we considered the principles outlined in the recently published PROBAST+AI guideline for evaluating prediction models (33). While our study’s primary aim was prognostic factor assessment rather than formal model development for clinical deployment, this framework provides a useful lens for self-evaluation.

Regarding participants and data sources, the single-center, retrospective design and the extended recruitment period (2000–2023) represent key limitations. Although we adjusted for diagnosis year and found no material impact on the main findings, unmeasured temporal changes in diagnostic criteria or treatment paradigms may still affect the generalizability of our results to other populations or contemporary practice. Regarding predictors, we included a comprehensive set of clinicopathological variables; however, Ki67 assessment may have been subject to variability over time, and granular treatment details (e.g., specific chemotherapy regimens) were unavailable for inclusion. The outcome measure (OS) was objectively defined and complete, which strengthens the study’s internal validity.

On the analytical front, we employed both conventional (Cox with bootstrap validation) and machine learning (RSF with OOB evaluation) methods, each with appropriate internal validation techniques for their respective frameworks. Nevertheless, external validation in independent cohorts—a critical requirement for any prediction model—was not performed and remains an essential direction for future research. Additionally, the absence of formal hyperparameter optimization for the RSF model, while acceptable given our focus on variable importance rather than maximized predictive accuracy, represents a methodological consideration for future studies aiming to optimize model performance.

Taken together, the applicability of our findings is most directly relevant to young women (≤40 years) with localized breast cancer treated in similar clinical settings. These PROBAST+AI-informed considerations should guide the interpretation of our results and reinforce the need for prospective multicenter studies to validate and refine these prognostic associations.

## 5 Conclusion

This study investigated prognostic factors in young breast cancer patients using both traditional and machine learning approaches. Invasive pathology, nipple invasion, and advanced T stage were associated with poorer survival, while endocrine therapy and radiotherapy were associated with better survival. The association of high Ki67 expression with better survival in this young cohort is notable and, given the potential for confounding by subtype and treatment patterns, warrants further investigation in well-designed prospective studies. The RSF model identified these as key predictors, highlighted radiotherapy as the most influential variable, and effectively stratified patients into distinct risk groups. These findings suggest a complex prognostic interplay in young patients and highlight the potential value of integrated analytical methods. Given the retrospective, single-center design and the long study period, further multicenter studies are needed to confirm these results and refine risk stratification for this population.

## Data Availability

The raw data supporting the conclusions of this article will be made available by the authors, without undue reservation.
