# Supplementary material for: Integrating machine learning and clinicopathological data to stratify survival risk in young women with localized breast cancer
Source: Front Med (Lausanne). 2026 May 5;13:1793790. doi: 10.3389/fmed.2026.1793790 (PMC13183654; doi:10.3389/fmed.2026.1793790)
Supplement: Supplementary file 1 [file Table_1.DOCX]

**Supplementary material**

**Supplementary Table S1.** Multivariable Cox regression analysis with adjustment for diagnosis year

| **Variables** | **HR (95% CI)** | **P value** |
| --- | --- | --- |
| Age (>32 vs. ≤32 years) | 0.73 (0.46-1.13) | 0.118 |
| T stage (T2-4 vs. Tis/T1) | 1.55 (1.01-2.45) | 0.038 |
| N stage (N1-3 vs. N0) | 1.54 (0.92-2.61) | 0.066 |
| Surgery (Mastectomy vs. BCS) | 0.76 (0.42-1.32) | 0.452 |
| Pathology (Invasive vs. Non-invasive) | 4.20 (1.17-20.10) | 0.048 |
| Ki67 (>35% vs. ≤35%) | 0.45 (0.32-0.86) | 0.004 |
| Nipple invasion (Yes vs. No) | 3.82 (2.02-6.85) | **<0.001** |
| Chemotherapy |  |  |
| Neoadjuvant vs. None | 1.96 (0.93-4.13) | 0.075 |
| Adjuvant vs. None | 1.52 (0.89-2.60) | 0.124 |
| Radiotherapy (Yes vs. No) | 0.60 (0.42-0.91) | **0.023** |
| Endocrine therapy (Yes vs. No) | 0.51 (0.29-0.71) | **0.004** |
| **Diagnosis year** |  |  |
| 2000-2004 | Reference |  |
| 2005-2009 | 0.98 (0.60-1.60) | 0.932 |
| 2010-2014 | 0.92 (0.55-1.54) | 0.748 |
| 2015-2019 | 0.87 (0.50-1.51) | 0.618 |
| 2020-2023 | 0.79 (0.42-1.49) | 0.464 |

Model adjusted for all listed variables and diagnosis-year categories.

**Abbreviations:** HR, hazard ratio; CI, confidence interval; BCS, breast-conserving surgery.
